# Etiology of Kidney Diseases With Proteinuria in the Gambia/West Africa

**DOI:** 10.3389/fped.2022.854719

**Published:** 2022-03-21

**Authors:** Udo Vester, Augustin Fombah, Maite Hölscher, Danlami Garba, Mary Tapgun, Pamela Collier N‘Jai, Philipp Mendy, Gibril Bass, Abdul K. Muhammad, Suzanne T. Anderson, Abdoulie Sanneh, Charles Onyeama, Udo Helmchen, Khalifa Bojang, Peter F. Hoyer, Tumani Corrah

**Affiliations:** ^1^Helios Klinikum St Johannes, Duisburg, Germany; ^2^Medical Research Council, Fajara, Gambia; ^3^University Children's Hospital, Kinderheilkunde II, University of Duisburg-Essen, Essen, Germany; ^4^Imperial College London, London, United Kingdom; ^5^Kidney Pathology Registry, University Hospital Hamburg, Hamburg, Germany

**Keywords:** kidney, proteinuria—nephrotic syndrome, kidney biopsy, histology, West Africa

## Abstract

In West Africa, kidney diseases are frequently seen, but diagnostic and therapeutic options are poor due to limited access to specialized facilities. To unravel the etiology and develop clinical guidelines, we collected clinical data and results of kidney biopsies in 121 pediatric and mostly young adult patients with edema and proteinuria in The Gambia. Workup included clinical examination, urine and serum analysis, and kidney biopsy findings. Selected cases were treated with steroids.

**Results:**

The median age was 14.9 years (range 1.8–52.0) at presentation. The most frequent underlying histologies were post-infectious glomerulonephritis (PIGN) in 38%, focal-segmental glomerulosclerosis (FSGS) in 30%, minimal change nephrotic syndrome (MCNS) in 15%, and membranous glomerulonephritis (MGN) in 10% of cases. Patients with PIGN were significantly younger and had less proteinuria and higher serum albumin levels than the other three. Infected scabies was seen more often in cases with PIGN. Clinical parameters could not distinguish patients with FSGS, MCNS, and MGN. Steroid response was prompt in patients with MCNS (remission in 10/10 cases) compared to FSGS (4/19) and MGN (0/4). In summary, the clinical histopathological correlation allows a better approach to therapy and can be the basis for urgently needed interventional studies in steroid-resistant cases.

## Introduction

In the public view, tropical infections represent the main medical problems in Africa. However, non-communicable diseases are increasingly recognized as a substantial health care burden (1), and kidney diseases seem to be especially frequent in Africa (2, 3). Apart from hypertension, glomerular diseases with proteinuria are the leading cause of kidney dysfunction (4). In contrast to the medical needs, diagnostic facilities and treatment for kidney diseases are only partially available in a minority of African countries (5).

In the African continent, the etiology of proteinuric kidney disease is known to be diverse and does include infections like HIV (6), hepatitis B (7), schistosomiasis (8), malaria (9), and streptococci (10). Besides infections, genetic factors have been suspected, as in the USA, where the Afro-American population has a higher prevalence and has a more severe kidney disease than other ethnicities (11). A linkage of kidney disease in the Afro-American population with some gene loci has been described (12).

In The Gambia, we have previously described a substantial number of children who presented with edema and proteinuria with significant morbidity (13). As no conclusive data from other West-Africa sites were available, therapeutic decisions based on the histopathology and etiology were not possible. Therefore, the MRC Fajara health care team requested biopsy support, which was not established in a country with limited resources. This study aimed to describe and analyze the dataset regarding the underlying kidney pathology and develop recommendations for better medical approaches and therapeutic management.

## Patients And Methods

Starting 2004, a total of 121 patients from various health care centers of the country admitted with both edema and proteinuria were included in this analysis. As locally available, clinical and laboratory workup consisted of a detailed history, physical examination, urine dipstick for hematuria and proteinuria, measurement of serum creatinine, total protein, albumin, and serology for HIV and hepatitis B surface antigen. Malaria was excluded with a microscopic blood film examination. Glomerular filtration rate was estimated (eGFR) using the Schwartz formula in children (14) and the Cockroft-Gault formula (15) in adult patients. INR (international normalized ratio of the prothrombin time) and platelets were measured prior to kidney biopsy. In a subgroup of patients, frozen serum samples were shipped to Europe, and complement C3c, anti-streptolysin-O (ASOT), and anti-DNAse titers were measured. A blood pressure value of more than 140/90 mmHg in adults and more than 120/80 mmHg in children below 14 years was regarded as hypertensive. After a detailed explanation by local staff members, every patient or their parent/legal guardian gave informed consent for biopsy and medical treatment. Kidney biopsies were done under ultrasound guidance and sedation with midazolam and ketamine by two experienced, board-certified pediatric nephrologists (UV and PH). Biopsy specimens were immediately fixed in buffered formalin and analyzed with light microscopy, immunostaining, and electron microscopy in Germany (UH). After the biopsy, patients were kept under close inpatient observation for 24 h. Conservative treatment using diuretics or antihypertensives was commenced as clinically indicated. Prednisone (1–2 mg/kg body weight) was introduced in the absence of contraindications in cases with assumed idiopathic nephrotic syndrome.

Statistical analysis was done using Stata12. As normal distribution of age was excluded using a skewness and kurtosis test, an alternative, K-sample equality of medians test, was used to find differences between subgroups with different kidney histology. A Chi-square test was used to find associations between kidney histopathology and clinical parameters. Fisher's exact test was used instead in cases where expected cell frequencies were <5. *P*-values of <0.05 were regarded significant. Data were expressed as the median and interquartile range (IQR).

## Results

### Patient Characteristics

**Figure 1:**
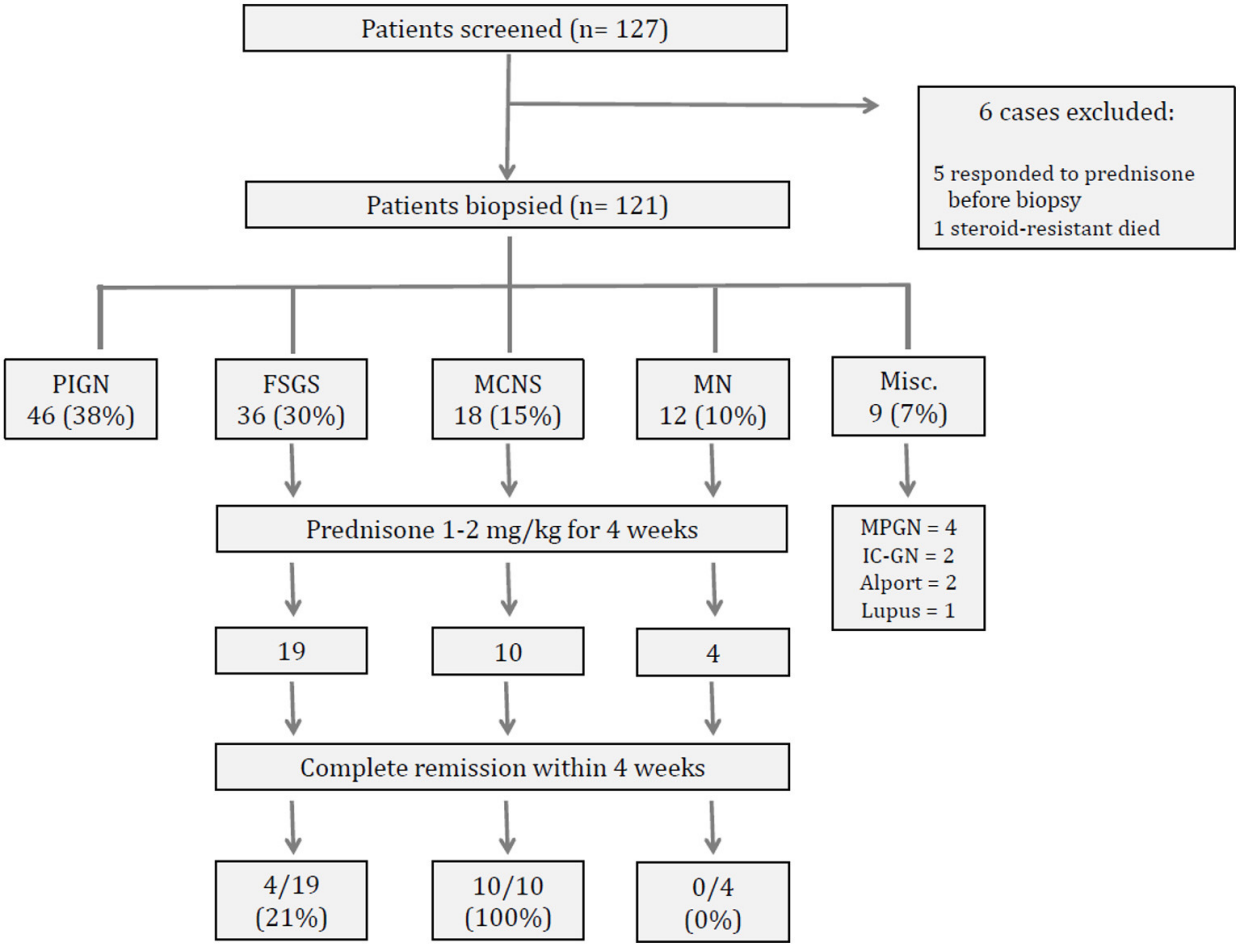
Flow chart of study population, biopsy results and response to prednisone.

**Figure 2:**
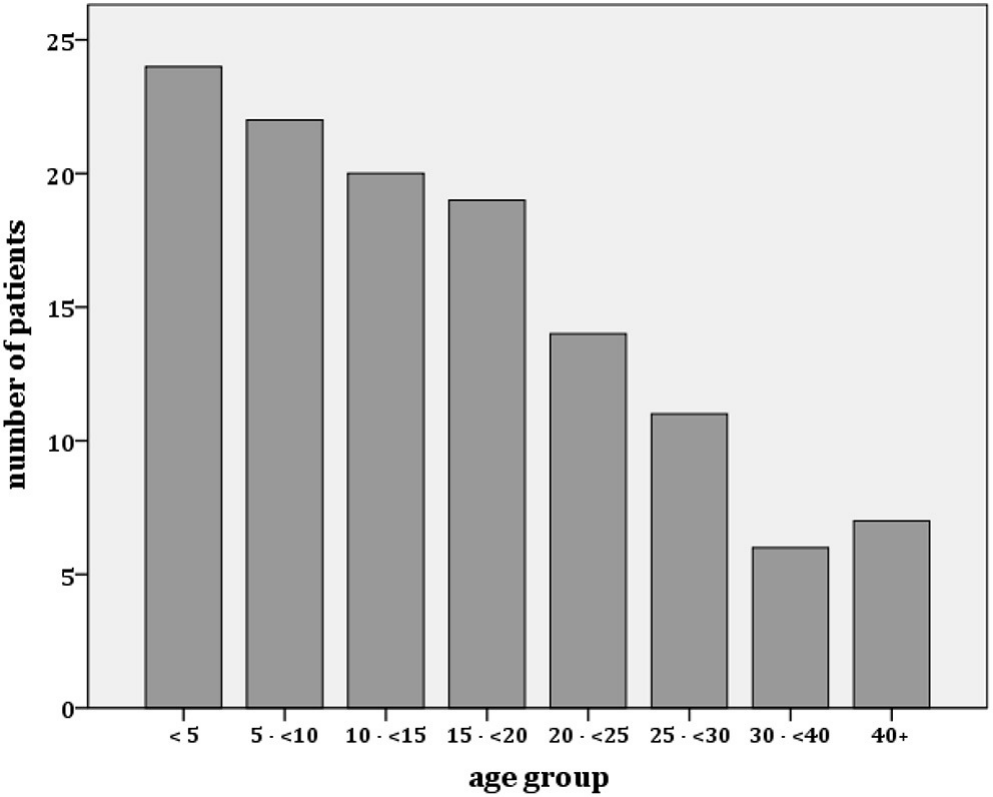
Age distribution of patients with proteinuric kidney disease in The Gambia.

A total of 127 patients were initially scheduled for biopsy (Figure 1). Six cases were excluded (five responded promptly to a 4-week course of prednisone and one patient with steroid-resistant nephrotic syndrome case died from septicemia before biopsy), leaving a total of 121 patients to be included in this analysis. The female/male ratio was 0.7 (50 female and 71 male). Median age at disease onset was 14.9 (IQR 16.5 {7.5–24}, range 1.8–52 years). The age distribution is depicted in Figure 2. Overall, most patients were children or adolescents, with 63 % of all cases being younger than 18 years. Edema was the presenting symptom in every case, either generalized or as puffiness of the face. Signs of super infected scabies skin lesions were found in 25/121 (21%) patients. Gross proteinuria (dipstick 3+/4+) and hematuria were seen frequently in 90/116 patients (78%), and 90/117 (77%) of patients, respectively. Sixty out of 121 (50%) patients were hypertensive. Kidney function, expressed as eGFR, could be calculated in 101 patients and was at median 77 ml/min/1.73 m^2^ (IQR 62 {54–116}). Kidney dysfunction with a CKD stage III or worse (GFR <60 ml/min/1.73 m^2^) was observed in 28/101 (28%) cases. Complement factor C3c was measured in 72 cases and decreased values below the lower normal value of 0.8 g/l were seen in seven patients. Anti-streptolysin O titers (ASOT) above the limit of 400 IU/l were seen in 15/69 cases and Anti-DNAse values above the limit of 200 IU/l were seen in 47/69 cases. Kidney biopsy was performed without any complication except in one pediatric patient with a transient macrohematuria without the need for blood transfusion.

### Histopathology

Results of kidney histopathology are shown in Figure 1. The main diagnoses were PIGN (*n* = 46) followed by FSGS (*n* = 36), MCNS (*n* = 18) and MGN (*n* = 12). These four diagnoses accounted for more than 90% of all cases. Membranoproliferative glomerulonephritis (MPGN, *n* = 4), immune-complex nephritis (*n* = 2), Alport syndrome (*n* = 2) or lupus nephritis (*n* = 1) were seen only occasionally. Therefore, further comparative analysis of data was done with the four main histological diagnoses (PIGN, FSGS, MCNS, MGN).

### Clinical Data

The clinical and laboratory data of patients with PIGN, FSGS, MCNS or MGN are shown in Table 1. Patients with PIGN differ significantly from the others as they are much younger and more often have hematuria or hypertension and less proteinuria or hypoalbuminemia.

**Table 1:** Comparison of clinical and laboratory data between main histological diseases.

	***PIGN value***	***FSGS***	***MCNS***	***MN***	***p value***
Age (IQR) [years]	7.1 (4.0–12.0)	19.9 (14.1–26.5)	20.1 (14.9–24.3)	19.0 (15.0–32.6)	<0.001^†^
Hematuria	43/43 (100%)	23/36 (63.9%)	9/18 (50%)	9/11 (81.8%)	<0.001
Hypertension	30/46 (65.2%)	13/36 (36.1%)	5/18 (27.8%)	5/12 (41.7%)	0.014
Proteinuria >2+	22/40 (55.0%)	31/36 (86.1%)	18/18 (100%)	11/12 (91.7%)	<0.001
Albumin <25g/l	7/40 (17.5%)	29/34 (85.3%)	16/16 (100%)	8/11 (72.7%)	<0.001
eGFR <60 ml/min/1.73m^2^	13/38 (34.2%)	9/30 (30.0%)	0/17 (0.0%)	3/9 (33.3%)	0.021^‡^^Φ^
Signs of scabies	19/46 (41.3%)	2/36 (5.6%)	1/18 (5.6%)	1/12 (8.3%)	<0.001^‡^

*Unless otherwise stated there is no statistically significant difference between FSGS, MCNS, and MN and the default test is a Chi-square*,

† *K-sample equality of medians test used*.

‡ *P value using Fishers exact test because some cell expected values are below 5*.

Φ *Significance from MCNS only*.

Anti-DNAse titers above the threshold of 200 IU/l were seen in 24/26 (92%) cases with PIGN; this is significantly more often than in patients with FSGS, MCNS, or MGN 23/37 (62%), *p* <0.022, Chi-square). In addition, ASOT above 400 IU/l were seen in 7/26 cases (27%) with PIGN compared to 3/38 (8%); however, this difference did not reach statistical significance (*p* = 0.116). Additionally, signs of recent scabies skin infection were seen significantly more often in cases with PIGN.

Complement C3c was decreased in 4/26 (15%) cases with PIGN with 0/43 with FSGS, MCNS, or MGN (n.s.). In addition, decreased C3c values were observed in two cases with MPGN and one with lupus nephritis.

Patients with FSGS, MCNS, or MGN could not be distinguished from each other by clinical parameters (Table 1). The majority of these cases were either adolescents or young adults and, in most cases, presented with gross edema, severe proteinuria, and hypoalbuminemia. The only significant difference observed between these three groups was the absence of impaired eGFR (<60 ml/min/1.73 m^2^) in cases with MCNS. In contrast, one-third of FSGS or MGN cases presented initially with impaired kidney function.

### Therapeutic Intervention

In 33 cases presenting as nephrotic syndrome [as defined as gross proteinuria > 300 mg/dl (dipstick 3+/4+) and serum albumin <25 g/l], the response to 4 weeks of treatment with prednisone 1–2 mg/kg could be evaluated (Figure 1). Overall, the positive response rate was 14/33 (42%). All patients with MCNS responded with complete remission within 4 weeks. This response rate was significantly higher than in patients with FSGS or MGN (Chi-square, *p* <0.01). All patients who responded to prednisone had a normal kidney function; whereas four cases of the non-responders had a eGFR below 60 ml/min/1.73 m^2^ at onset (Chi-square, *p* <0.05).

In addition, prednisone response could be evaluated in the six cases scheduled for biopsy. Five patients responded rapidly to prednisone and, therefore, did not undergo a kidney biopsy. A 20-year-old male patient died from streptococcal septicemia a few days before he was scheduled for a kidney biopsy. Thus, the overall steroid response increased to 19/39 (49%) with these patients included.

Three cases with FSGS, not treated with prednisone, had spontaneous remission. All were male, aged 17, 22, and 27 years and presented initially with anasarca, a serum albumin concentration of 13–15 g/l, and normal eGFR.

### Infections

Three patients were HBsAg positive (FSGS in two, MGN in one), and one patient tested positive for HIV-1 infection (FSGS). None received treatment with steroids.

Six patients, all with ascites, presented with spontaneous bacterial peritonitis (FSGS in four cases, MCNS and MGN one each), and streptococcus pneumoniae was cultured in the blood of five of them.

## Discussion

We found a wide range of kidney diseases in our series of 121 patients from The Gambia biopsied because of edema and proteinuria. In more than 90% of cases, one of the four most prevalent diseases could be detected: PIGN, FSGS, MCNS, and MGN. Other diagnoses like MPGN, Lupus-Nephritis, or Alport-Syndrome were seen only occasionally. From these data, we can generate several observations.

Patients with PIGN mainly present in childhood, usually with an acute nephritic syndrome defined as moderate proteinuria, hypertension and/or hematuria or kidney impairment (16). It has been argued that the main reason for PIGN in the developing world is streptococcal skin infection (16), and this has been regularly associated with scabies (10, 17). In our patients from The Gambia, scabies and raised ASOT or anti-DNAse titers were more often seen in patients with PIGN, strongly indicating a role of streptococcal skin infection. Complement C3c was decreased only in a subgroup of patients with PIGN, but it can be speculated that C3 consumption would have been more prevalent if blood samples had been drawn earlier in the course of the disease (16).

It is estimated that PIGN is subclinical in most cases, and therefore the true prevalence of acute nephritis is likely to be much higher (16). Although regarded as a primarily benign condition, recent data suggest a significant contribution to kidney disease later in life (18). With these sequelae in mind, scabies “may have an impact beyond that of an annoying skin problem” (19), and therefore, may justify eradication efforts. Further field studies are required to elucidate the true magnitude of scabies, super-infected scabies, and acute nephritic syndrome in West Africa.

In The Gambia, patients with nephrotic syndrome present mainly as adolescents or young adults, and FSGS, MCNS, and MGN could not be differentiated by clinical, or laboratory means. Similar to other African countries (20), FSGS is the most prevalent nephrotic disease in The Gambia. The reason for this remains obscure as infections like hepatitis or HIV present in just a small subgroup of patients. A genetic influence has been postulated as FSGS is seen more often in African Americans, (11) and the African population of South Africa (20). In the Caucasian populations, many genes have been identified in the last two decades, coding for podocyte structures like *NPHS1, NPHS2*, and *WT1*, being the most frequent ones (21). However, these genes seem to be of minor importance in African American patients (22). Recent studies have shown a positive correlation between *MYH9* and *APOL1* polymorphisms and kidney disease (12). This association is not fully understood but may have evolved as a protective genetic variant against trypanosome infection (12). Circulating permeability factors have recently been associated with idiopathic FSGS (23). However, their role has not been studied in Africa so far.

In European children, distinguishing between hereditary and idiopathic forms of FSGS is important as this predicts the response to immunosuppressive treatment (24). In our Gambian patients with FSGS, four out of 19 cases (21%) responded to a 4-week course of prednisone, indicating that at least a subgroup of these patients has a responsive disease. Another three patients showed spontaneous remission, a phenomenon rarely seen in FSGS and not fully understood (25). For those steroid-resistant cases, further studies in West Africa are urgently needed to elucidate the role of other treatment options like cyclosporine (26) and progression-slowing conservative therapy, e.g., with angiotensin-converting-enzyme inhibitors (ACEI).

MCNS was seen in a substantial number of our patients. Generally, MCNS is regarded as a disease of early childhood in the northern hemisphere (27) and rarely occurs in African patients (28). In contrast, in The Gambia, MCNS was observed quite frequently in young adults with a median age of 20.1 years. We speculate that these patients represent a particular subgroup that deserves further attention. The alternative explanation that younger children were not presenting to our health facility seems unlikely as children with PIGN and much less edema regularly attend. It is of note that all cases with MCNS showed normal eGFR, and those who were treated with prednisone responded promptly to a 4-week course. However, the risk of relapse is unknown, needs to be defined and strategies for long-term treatment developed.

MGN was seen at the same age as patients with FSGS or MCNS. MGN is often associated with chronic infections like hepatitis and malignancies; however, the vast majority of cases are “idiopathic” (29). It is well known that MGN harbors the potential for spontaneous resolution, but it may also progress to end-stage kidney failure, making treatment decisions difficult (29). It is of note that vaccination programs against hepatitis B may affect the prevalence of hepatitis-B-associated MGN.

Recently, autoantibodies against Phospholipase A2-receptor have been described to play a major role (30). Again, experience with long-term follow-up in these patients is needed to clarify prognosis and treatment options in The Gambia.

The study's weakness is that at this stage, it is explorative, describing the clinical presentation in relation to the pathohistology with no control data. Long-term follow-up was hampered by the evolving Ebola crisis and later, the corona pandemic.

**Figure 3:**
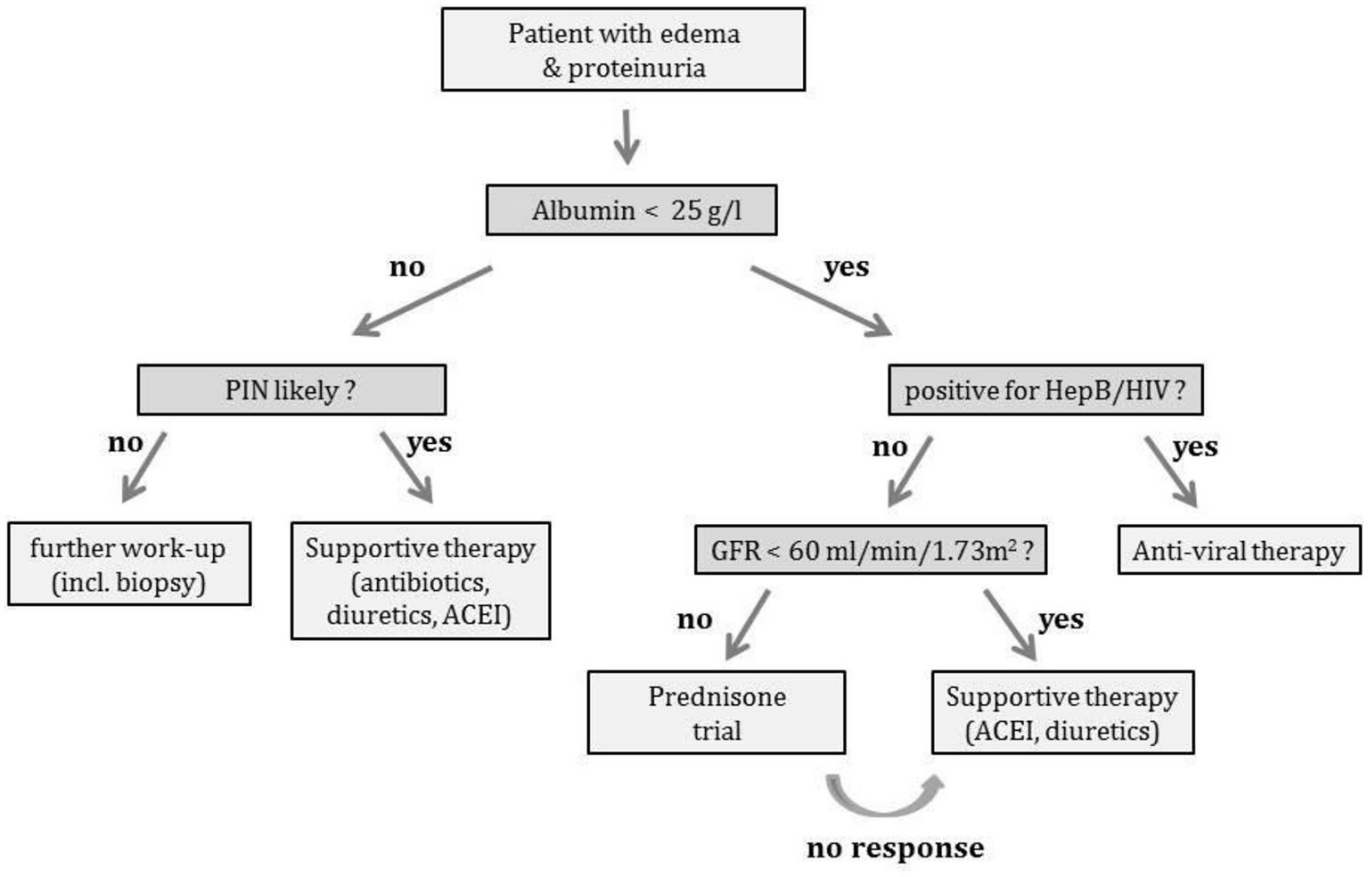
Work-up algorithm. Work-up of patients with proteinuria (* a prednisone trial, if a RPGN seems unlikely; supportive therapy includes diuretics, antibiotics, anti-hypertensives and anti-proteinuric medication with angiotensin converting enzyme inhibitors).

In conclusion, proteinuric kidney diseases represent a frequent medical problem in The Gambia West Africa. For the first time we were able to describe underlying etiologies. Acute nephritic syndrome with moderate proteinuria is mainly a childhood disease and histopathology characterized as PIGN. It is speculated that super-infected scabies precipitates most cases. These patients need supportive treatment only as most episodes will resolve. However, the long-term sequelae are unclear and subject to concerns. Nephrotic syndrome with hypoalbuminemia is mainly due to FSGS. However, a substantial number of patients do have MCNS. Therefore, a steroid trial seems to be justified after exclusion of HIV or hepatitis B virus infection. Further studies should elucidate the role of candidate genes like *APOL1* (12) and focus on treatment options in steroid-resistant cases. As a first step to improved care, we propose from our data the diagnostic and therapeutic algorithm shown in Figure 3.

We certainly feel it is time to offer the benefits of clinical nephrology to the large number of patients with proteinuric kidney diseases in West Africa. As kidney replacement therapy is hardly available for West African patients, developing simple but effective therapeutic options is of utmost importance. Furthermore, our data may be beneficial for designing future interventional trials, hopefully, possible in the post corona pandemic age.

## Data Availability Statement

The original contributions presented in the study are included in the article/supplementary material, further inquiries can be directed to the corresponding author.

## Ethics Statement

The studies involving human participants were reviewed and approved by MRC Laboratories, Fajara, The Gambia. Written informed consent to participate in this study was provided by the participants' legal guardian/next of kin.

## Author Contributions

UV and PH have made substantial contributions to the conception or design of the work, or the acquisition, analysis, or interpretation of data for the work, drafted the work and revised it critically for important intellectual content, have approved the final version to be published. UV, PH, and TC agree to be accountable for all aspects of the work in ensuring that questions related to the accuracy or integrity of any part of the work are appropriately investigated and resolved. TC has the final clinical responsibly for the whole project, revised it critically for important intellectual content, has approved the final version to be published. All other persons have made substantial contributions to the work reported in the manuscript, including those who provided editing and writing assistance and all authors agree to be accountable for the content of the work. All authors contributed to the article and approved the submitted version.

## Funding

This study was supported by research funds from the Klinik für Kinderheilkunde II, University Children‘s Hospital Essen/Germany, by grants from the Forschungsunterstützungskreis Kindernephrologie Essen e.V. and from the Herrmann-May-Stiftung of the German Society for Pediatrics (DGKJ e.V).

## Conflict of Interest

The authors declare that the research was conducted in the absence of any commercial or financial relationships that could be construed as a potential conflict of interest.

## Publisher's Note

All claims expressed in this article are solely those of the authors and do not necessarily represent those of their affiliated organizations, or those of the publisher, the editors and the reviewers. Any product that may be evaluated in this article, or claim that may be made by its manufacturer, is not guaranteed or endorsed by the publisher.
